# Effectiveness of a Behavior Change Technique–Based Smartphone Game to Improve Intrinsic Motivation and Physical Activity Adherence in Patients With Type 2 Diabetes: Randomized Controlled Trial

**DOI:** 10.2196/11444

**Published:** 2019-02-13

**Authors:** Christoph Höchsmann, Denis Infanger, Christopher Klenk, Karsten Königstein, Steffen P Walz, Arno Schmidt-Trucksäss

**Affiliations:** 1 Department of Sport, Exercise and Health University of Basel Basel Switzerland; 2 Pennington Biomedical Research Center Baton Rouge, LA United States; 3 Centre for Design Innovation Swinburne University of Technology Melbourne Australia

**Keywords:** behavior change, exercise adherence, gamification, intrinsic motivation, mhealth, mobile phone game, physical activity, type 2 diabetes

## Abstract

**Background:**

Regular physical activity (PA) is an essential component of a successful type 2 diabetes treatment. However, despite the manifest evidence for the numerous health benefits of regular PA, most patients with type 2 diabetes remain inactive, often due to low motivation and lack of PA enjoyment. A recent and promising approach to help overcome these PA barriers and motivate inactive individuals to change their PA behavior is PA-promoting smartphone games. While short-term results of these games are encouraging, the long-term success in effectively changing PA behavior has to date not been confirmed. It is possible that an insufficient incorporation of motivational elements or flaws in gameplay and storyline in these games affect the long-term motivation to play and thereby prevent sustained changes in PA behavior. We aimed to address these design challenges by developing a PA-promoting smartphone game that incorporates established behavior change techniques and specifically targets inactive type 2 diabetes patients.

**Objective:**

To investigate if a self-developed, behavior change technique-based smartphone game designed by an interdisciplinary team is able to motivate inactive individuals with type 2 diabetes for regular use and thereby increase their intrinsic PA motivation.

**Methods:**

Thirty-six inactive, overweight type 2 diabetes patients (45-70 years of age) were randomly assigned to either the intervention group or the control group (one-time lifestyle counseling). Participants were instructed to play the smartphone game or to implement the recommendations from the lifestyle counseling autonomously during the 24-week intervention period. Intrinsic PA motivation was assessed with an abridged 12-item version of the Intrinsic Motivation Inventory (IMI) before and after the intervention. In addition, adherence to the game-proposed PA recommendations during the intervention period was assessed in the intervention group via the phone-recorded game usage data.

**Results:**

Intrinsic PA motivation (IMI total score) increased significantly in the intervention group (+6.4 (SD 4.2; *P*<.001) points) while it decreased by 1.9 (SD 16.5; *P*=.623) points in the control group. The adjusted difference between both groups was 8.1 (95% CI 0.9, 15.4; *P*=.029) points. The subscales “interest/enjoyment” (+2.0 (SD 1.9) points, *P*<.001) and “perceived competence” (+2.4 (SD 2.4) points, *P*<.001) likewise increased significantly in the intervention group while they did not change significantly in the control group. The usage data revealed that participants in the intervention group used the game for an average of 131.1 (SD 48.7) minutes of in-game walking and for an average of 15.3 (SD 24.6) minutes of strength training per week. We found a significant positive association between total in-game training (min) and change in IMI total score (beta=0.0028; 95% CI 0.0007-0.0049; *P*=.01).

**Conclusions:**

In inactive individuals with type 2 diabetes, a novel smartphone game incorporating established motivational elements and personalized PA recommendations elicits significant increases in intrinsic PA motivation that are accompanied by de-facto improvements in PA adherence over 24 weeks.

**Trial Registration:**

ClinicalTrials.gov NCT02657018; https://clinicaltrials.gov/ct2/show/NCT02657018

## Introduction

Diabetes mellitus affects over 400 million adults worldwide [1], and 90%-95% of all cases are attributed to type 2 diabetes [2]. The disease is associated with numerous complications and comorbidities that drastically increase direct and indirect medical costs and considerably contribute to the disease’s enormous financial strain worldwide [3].

Regular physical activity (PA), with its proven positive effects on glucose and lipid metabolism, blood pressure, cardiovascular complications, and quality of life, is a key component of successful diabetes treatment [4]. Despite these health benefits and the fact that patients with diabetes are usually encouraged to increase PA by their physicians, long-term adherence to PA-promoting programs is generally poor, and the level of regular PA remains low [2,5]. PA has been estimated to be responsible for 6%-10% of the world’s type 2 diabetes prevalence [6] and increase the risk of all-cause mortality by an estimated 60% [7]. Lack of infrastructure, missing social support, health concerns, and especially low motivation and lack of PA enjoyment are the main deterrents that keep patients with type 2 diabetes from effectively changing their PA behaviors [8,9]. As intrinsically motivated individuals have been shown to have higher PA engagement and better PA adherence than those who are primarily motivated by external factors [10], PA-promoting programs should aim at particularly helping patients increase their PA enjoyment, and consequently, intrinsic motivation for regular PA [11].

A promising approach that has increasingly been examined in recent years to promote regular PA in unmotivated, inactive target groups is exergames. Through the enjoyable game experience, console-based exergames have been shown to motivate inactive patients with type 2 diabetes to voluntarily engage in more regular PA and thereby improve their glycemic control and overall health status [12]. Very recently, PA-promoting game apps such as Pokémon GO (Niantic Labs, San Francisco, CA) have entered the market and likewise aim at sustaining PA habits through gamified incentives. Although Pokémon GO certainly has the potential to increase daily PA, it should be noted that the game-related initial increases of daily PA of 25% in the first week have been shown to gradually diminish in subsequent weeks and return to baseline after only 6 weeks [13]. It is possible that the game design does not include a sufficient degree of narrative, gameplay, or storytelling, which are required to sustainably motivate the player to play the game and consequently make a PA-promoting game effective in the long term [14]. To address these design challenges, with an interdisciplinary team featuring sports scientists, gamification researchers, professional game developers, and clinical professionals, we developed a novel smartphone game that incorporates established motivational elements [15] and behavior change techniques [16] to encourage inactive patients with type 2 diabetes to adopt a healthier, more active lifestyle.

The purpose of this study was to investigate if the behavior change technique–based smartphone game can motivate inactive individuals with type 2 diabetes for regular use and thereby increase their intrinsic PA motivation. We hypothesized that use of the game would lead to greater improvements in intrinsic PA motivation than a control intervention consisting of a one-time lifestyle counseling and thereby increase PA adherence.

## Methods

### Study Design

This 24-week randomized controlled trial was conducted in accordance with the Declaration of Helsinki [17] between August 2016 and April 2018 at the Department of Sport, Exercise and Health of the University of Basel, Switzerland (trial registration: NCT02657018) and was approved by the local ethics committee (EKNZ 2015-424). Written informed consent was obtained from all study participants prior to inclusion in the study. The primary aim of this study was to investigate the effect of a novel PA-promoting smartphone game on daily PA in inactive patients with type 2 diabetes, measured as steps per day with the previously validated Garmin Vivofit 2 activity wristband [18] for 1 week before and after the intervention period. A significant increase in daily PA was found in the intervention group with average postintervention step counts corresponding to established PA recommendations [19]. The increases in daily PA were accompanied by significant improvements in aerobic capacity and stabilization of the glycemic control, measured as hemoglobin A1c (C. Höchsmann, personal communication, June 2018). Additional aims were to assess the effect of the game on the predefined [20] further outcomes “intrinsic PA motivation” and “PA adherence.” Participants were allocated at random to either the intervention or the control group using R version 3.2.3 (R Foundation for Statistical Computing, Vienna, Austria) and the R add-on package “blockrand” version 1.3 to apply permuted block randomization with randomly varying block sizes. The randomization list was generated in advance and transmitted by a person not involved in the study by using serially numbered, sealed, opaque envelopes. All outcome assessors were blinded with respect to the group allocation. It has been shown that after the initial 6 weeks, adherence to an exercise program in patients with type 2 diabetes decreases steadily, with a dropout of almost 80% after 6 months but is relatively stable beyond the 6-month timeframe [21]. Therefore, we chose an intervention duration of 24 weeks, which matches this critical 6-month timeframe and would consequently allow assessment of the longer-term PA adherence. The detailed study protocol can be found in a previous publication [20].

### Recruitment

Physically inactive overweight (body mass index ≥ 25 kg/m^2^) patients with type 2 diabetes (noninsulin-dependent) between the ages of 45 and 70 years were recruited in cooperation with various hospitals, doctor’s offices, and diabetes care centers in the Basel metropolitan area and via online and newspaper advertising. Eligible participants were required to have used a smartphone regularly during the year before the study to ensure that they were technologically proficient enough to represent a realistic target audience for a PA-promoting smartphone game. During the eligibility screening, participants underwent a medical examination including height, body mass, body fat content (by bioelectrical impedance analysis), and resting blood pressure measurements as well as resting electrocardiography. To verify insufficient levels of PA before the study (<150 minutes of moderate-intensity PA per week), participants filled out the Freiburg Questionnaire of PA [22] as part of the eligibility screening. Present health risks that contraindicate exercise testing [23] as well as an impaired physical mobility, acute infections, or injuries excluded participants from enrolling and continuing their participation in the study.

### Intervention

After the baseline assessment, the self-developed and commercially released smartphone game “Mission: Schweinehund” was installed on the phones of participants in the intervention group. The game was designed to be self-explanatory and motivate for regular use through its game mechanics. Therefore, participants only received basic instructions regarding the game controls, which were not related to an intended frequency or duration of use during the intervention period. The game uses the self-determination theory as the theoretical framework [24]. The self-determination theory is a widely researched theory of motivation that addresses both intrinsic and extrinsic motives for acting and has shown its utility in explaining processes that underpin exercise behavior [25] as well as motivation to play video games [26]. The goal of the game is to restore a decayed garden by planting trees and flowers. In doing so, the player attracts animals that used to live in the garden to come back and help with the restoration process. At the same time, the Schweinehund, the game’s adversary, is kept in check. In German, “innerer Schweinehund” (inner swine hound) refers to the weak or lazy part of one’s nature, often regarding PA, that has to be overcome to get one’s self going. The garden setting was deliberately chosen, as its restoration stands metaphorically for the restoration of the player’s body through regular PA. In addition, it has been shown that gardening is among the target group’s preferred forms of PA [27] and that gardening-themed games are quite popular and comprehensible to a wide range of players because of their straightforward mechanics [28,29]. In the game, regular PA is rewarded with water or building materials that are needed to restore the garden and proceed in the storyline. When designing the game, close attention was paid to the inclusion of established motivational elements [15] and behavior change techniques [16]. In addition to the rewards for successful PA behavior, goal setting, action planning, feedback on performance, and prompts and cues were incorporated into the game mechanics to support sustained changes in PA behavior. During the development phase of the game, all design choices, behavioral/motivational elements, and game mechanics were tested extensively with the target group regarding usability and motivational efficacy. Overall, four user studies (unpublished data) with a total of 44 participants and various thematic foci (eg, motivational efficacy of game concept and storyline, suitability of in-game workout regimen and baseline tests, usability of sensor tracking, and suitability of motivational elements) were conducted during the 26-month development phase.

The game’s PA content includes in-game workouts as well as the promotion of daily PA that follow the American College of Sports Medicine and European Association for Cardiovascular Prevention & Rehabilitation principles of exercise training [30,31]. In-game workouts consist of 130 variations of strength, endurance, balance, and flexibility exercises whose execution, as well as daily PA, is tracked via the phone’s sensors (camera, accelerometer, and gyroscope). To allow individualization of the PA-related content of the game, exercise tests such as the 1-minute Sit-to-Stand Test [32] and the 6-Minute Walk Test [33] assess the player’s fitness level at baseline and periodically during play. Based on the results, an algorithm selects appropriate entry levels and tailored rates of intensity progression for the exercise regimens and the daily PA goals, which could also be manually adjusted by the player to fit personal preferences and potential physical limitations. An individualization of the exercise regimen is crucial because unrealistic, overwhelming targets often reduce patients’ motivation and thereby directly affect adherence [34]. Regularity of PA and relative improvements rather than high absolute values determine the progression in the game and thereby make game success independent of the individual fitness level. Participants in the control group received a one-time lifestyle counseling to promote baseline activities of daily life [35]. Further, control group participants were provided with a structured exercise plan consisting of strength and endurance exercises with moderately increasing intensity and duration, comparable to the content of the game that was to be implemented autonomously during the intervention period. A detailed description of the game including screenshots can be found in a previous publication [20].

### Outcome Measures

#### Intrinsic Physical Activity Motivation

Intrinsic PA motivation was measured using an abridged 12-item version of the Intrinsic Motivation Inventory (IMI) at baseline and after the 24-week intervention. The IMI has gained widespread acceptance as a multidimensional measure of intrinsic motivation in the context of sports and physical activity [36-41]. The questionnaire included four subscales: “interest/enjoyment,” “perceived competence,” “perceived choice,” and “value/usefulness.” These subscales have been previously used to assess participants’ subjective experience regarding participation in a television exercise program [36] and to examine the motivational pull of video games [26]. The “interest/enjoyment” subscale is considered the true self-report measure of intrinsic motivation, whereas “perceived competence,” “perceived choice,” and “value/usefulness” are viewed as positive predictors of intrinsic motivation [36]. The items of each subscale were modified to fit the content of this study and rated on a 7-point Likert scale ranging from 1 (*not at all true*) to 7 (*very true*). This yielded total scores between 3 and 21 for each subscale and between 12 and 84 for the entire questionnaire. Higher scores indicated more internally motivated, self-regulated PA behavior.

#### Physical Activity Adherence

In the intervention group, adherence to the game-proposed PA recommendations was assessed during the intervention period via the recorded usage data from the participants’ phones. Usage data included daily PA (steps per day), completed and canceled in-game workouts, and patterns and total duration of game use. The accuracy of iPhones and Android phones to detect steps during various walking conditions independent of the placement on the body has been confirmed in our previous study [18]. For in-game walking, stride cadence was measured to assess periods of moderate-to-vigorous-intensity walking defined as ≥100 steps/minute [19].

### Statistical Analysis

Summary statistics were calculated to characterize the study sample and for pre- and postintervention data, as appropriate. Continuous data were summarized using mean (SD) and median (interquartile range [IQR]). Intrinsic PA motivation (IMI total score and scores for all four subscales) after the intervention was analyzed by analysis of covariance [42]. Results are presented as differences in outcome (with 95% CI) between participants in the intervention group and those in the control group, adjusted for the corresponding values at baseline. PA adherence was analyzed descriptively using the median, IQR, and range to illustrate game-related and overall-recorded daily PA per week during the 24-week intervention period. A linear regression model was used to assess the relationship between total in-game training (minutes) and change in IMI total score. Assumptions of the analysis of covariance were checked visually using residual plots. All statistical methods used to compare the groups for intrinsic PA motivation in the present report were prespecified and registered. R 3.4.0 (R Foundation for Statistical Computing) was used for statistical analyses and graphics with the significance level set to .05 (two-sided).

### Sample Size

An *a priori* sample size calculation based on the primary outcome was conducted for this study. Since intrinsic PA motivation and PA adherence are secondary outcomes, the corresponding analyses presented in this report should be considered explorative, and we reported the evidence in the data for the hypothesized effect of the game use on intrinsic PA motivation and PA adherence.

## Results

### Participant Flow and Characteristics

**Figure 1:**
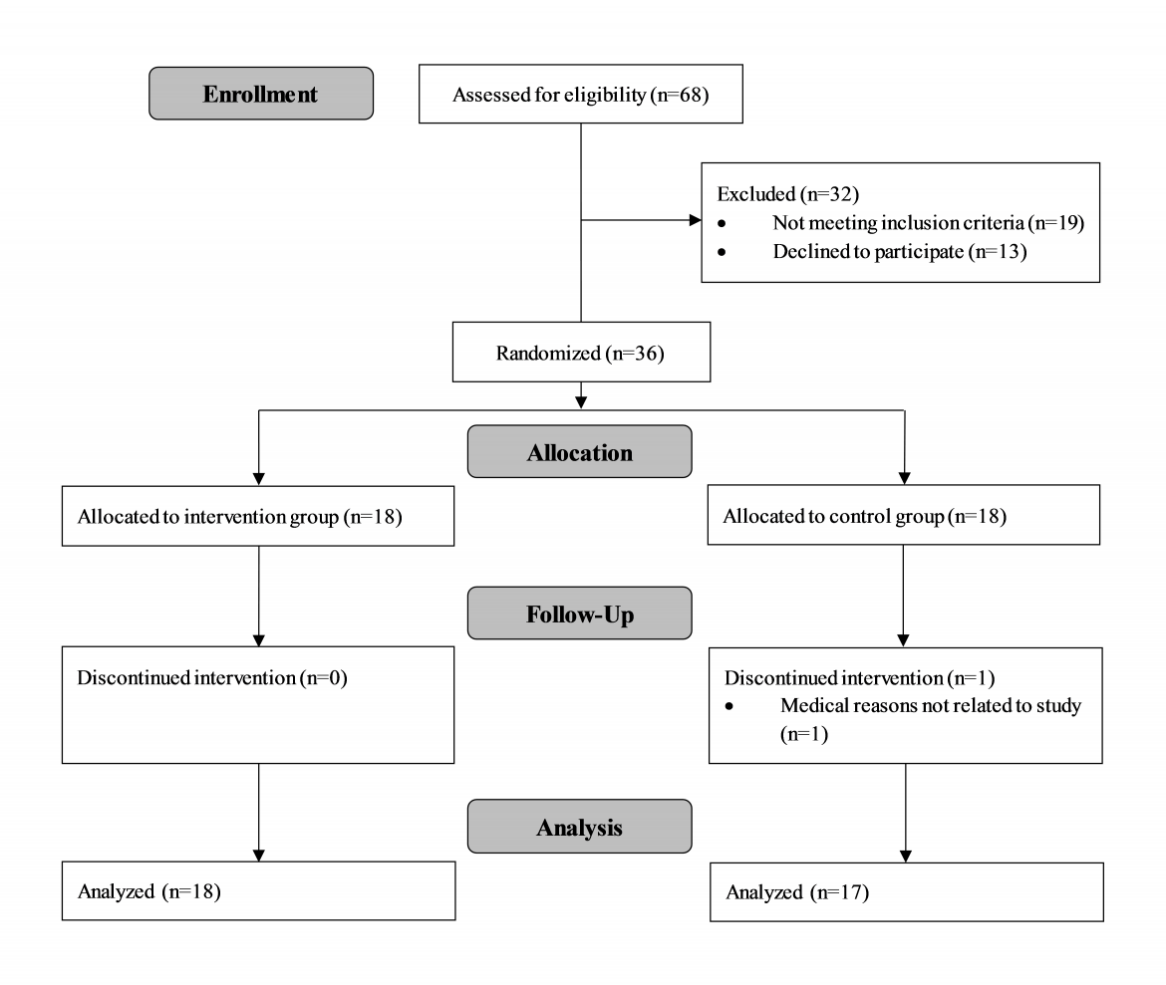
Flow diagram of study participants.

Figure 1 illustrates the participants’ flow through the study. A total of 68 subjects were assessed for eligibility, of which 19 did not meet the inclusion criteria and 13 declined to participate, leading to the exclusion of 32 subjects. All the remaining participants (n=36) were randomly assigned to either the intervention group (n=18) or the control group (n=18) (Figure 1). Baseline characteristics of study participants were balanced between the two groups (Table 1). One participant dropped out of the study before follow-up due to medical reasons not related to the study. No study-related or other adverse events were reported during the intervention period. Further, 35 participants completed the study and were included in the analyses.

### Changes in Intrinsic Physical Activity Motivation

**Figure 2:**
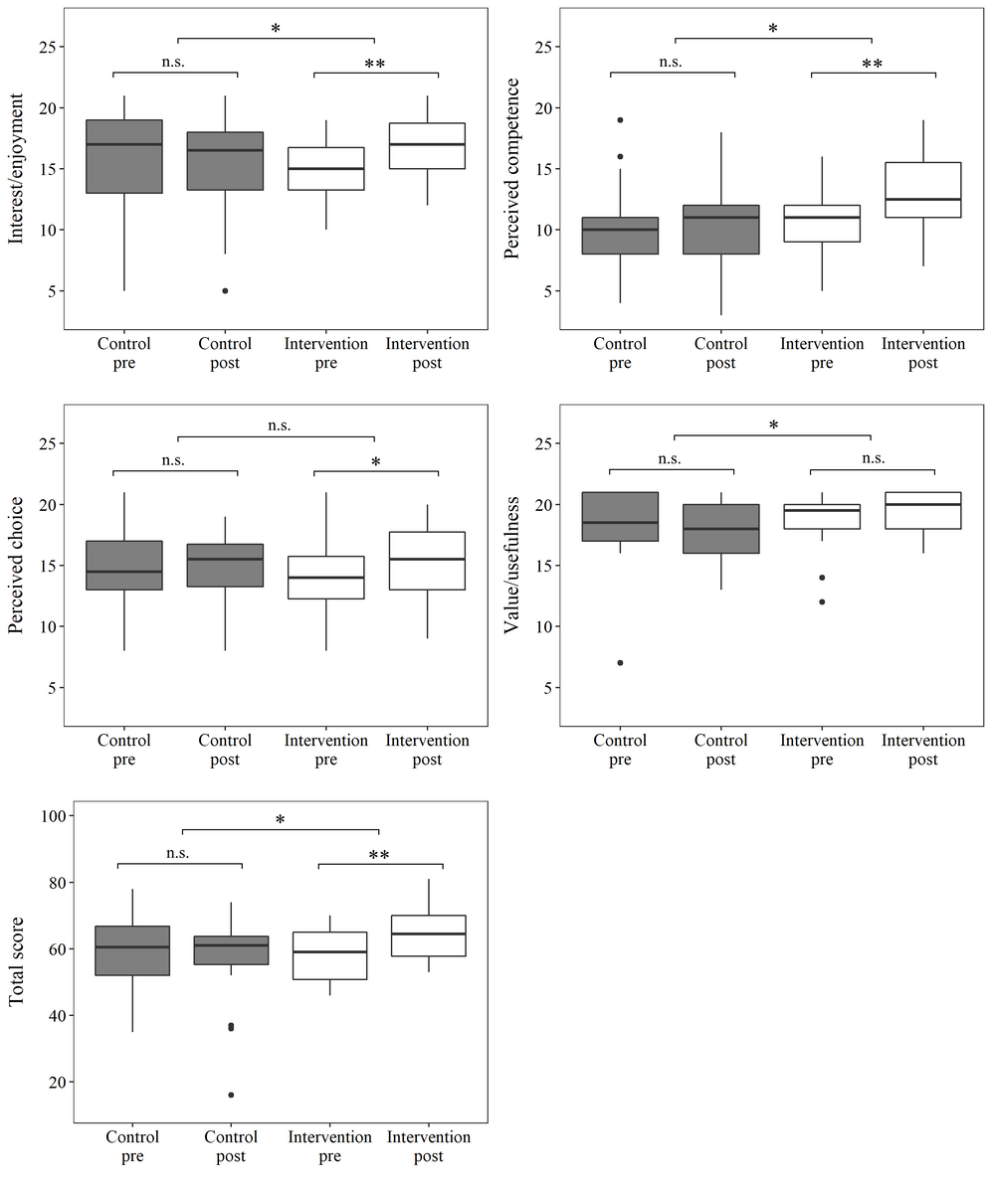
Illustration of the group-dependent change in total Intrinsic Motivation Inventory score and for the subscales "interest/enjoyment", "perceived competence", "perceived choice", and "value/usefulness". Pre: value at baseline; Post: value after the 24-week intervention period; n.s., not significant. *P<.05, **P<.001.

Figure 2 shows the pre- and postintervention data as mean and IQR for the IMI total score and all four subscales. Intrinsic PA motivation (IMI total score) increased significantly by an average of 6.39 (SD 4.19; *P*<.001) points in the intervention group and decreased by an average of 1.94 (SD 16.46; *P*=.62) points in the control group with an adjusted difference of 8.15 points (95% CI 0.90-15.39; *P*=.03) between the two groups. Similarly, we observed significant increases in scores for the subscales “interest/enjoyment” (by 2.00 [SD 1.94] points, *P*<.001) and “perceived competence” (by 2.44 [SD 2.36] points, *P<*.001) in the intervention group but no significant change in the control group. The adjusted difference between the two groups was 2.03 points (95% CI 0.04-4.09; *P*=.049) for “interest/enjoyment” and 2.88 points (95% CI 0.59-5.17, *P*=.02) for “perceived competence.” The value/usefulness subscale showed a significant adjusted difference of 2.72 points (95% CI 0.28-5.16; *P*=.03) in favor of the intervention group despite nonsignificant changes in either group. The score for the “perceived choice” subscale increased significantly by 1.22 (SD 2.44) points in the intervention group, but with a nonsignificant adjusted difference of 0.67 points (95% CI –1.27 to 2.61; *P*=.91) between the two groups.

**Table 1:** Baseline characteristics of study participants.

Characteristic		Intervention (n=18)		Control (n=18)	
		n	Mean (SD)	n	Mean (SD)
**Sex**					
	Female	8	—^a^	9	—
	Male	10	—	9	—
Age (years)		18	56 (5)	18	58 (6)
Height (m)		18	171 (7)	18	172 (8)
Weight (kg)		18	93 (12)	18	100 (16)
Body mass index (kg/m^2^)		18	32 (4)	18	34 (5)
Fat mass (%)		18	38 (7)	18	38 (7)
Moderate-to-vigorous physical activity (min/week)		18	39 (34)	18	37 (31)
Diabetes duration (years)		18	4 (4)	18	5 (4)

^a^Not applicable.

### Physical Activity Adherence

**Figure 3:**
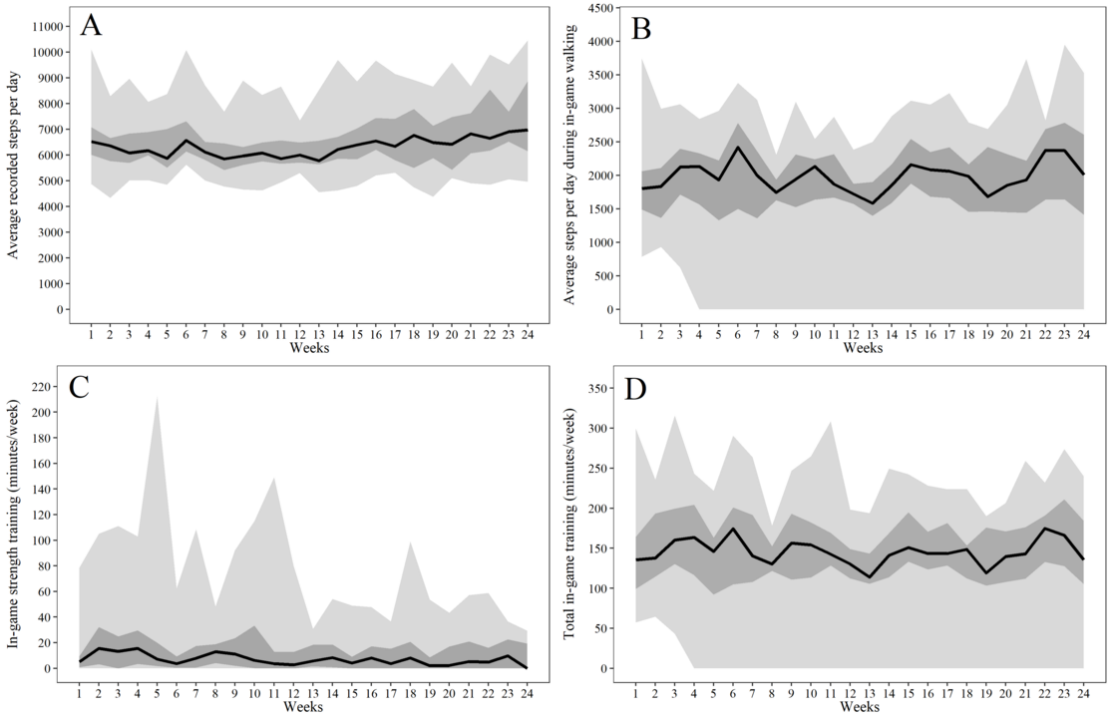
Illustration of phone-recorded physical activity data during the intervention period. A: weekly average of total steps per day, B: weekly average of steps per day during in-game training, C: average duration (minutes) of in-game strength training per week, D: total duration (minutes) of in-game training per week.

Figure 3 illustrates the usage data regarding daily PA as well as patterns and total duration of in-game training as median, IQR, and range. On an average, the participants’ phones recorded 6559 (SD 1182) steps per day during the 24-week intervention period. The weekly steps during in-game walking, averaged per day, are also shown in Figure 3, with an average of 1893 (SD 723) daily steps. Participants performed an average of 4.9 (SD 1.3) in-game walking trainings per week (average duration of 26.8 [SD 8.2] minutes per training), leading to a total average duration of 131.1 (SD 48.7) minutes of in-game walking per week. The analysis of the stride cadence revealed that on an average, 83.7% (SD 3.5%) of all in-game walking was done at a cadence of ≥100 steps/minute, indicating an average weekly amount of 109.8 (SD 43.4) minutes of moderate-to-vigorous-intensity in-game walking. One participant stopped playing the game after the third week and consequently did not produce any usage data beyond that time. We did not exclude this participant from the analyses because we consider nonuse as important of an outcome as regular use. Participants engaged in an average of 6.8 (SD 6.4) strength workouts per week with a total average duration of 15.3 (SD 24.6) minutes per week. In total, participants used the game for an average of 143.1 (SD 59.1) minutes of training per week. Overall 70.4% of strength workouts and 96.5% of walking workouts were completed, and 82.6% of all in-game workout reminders led to a completed work out on the same day.

**Figure 4:**
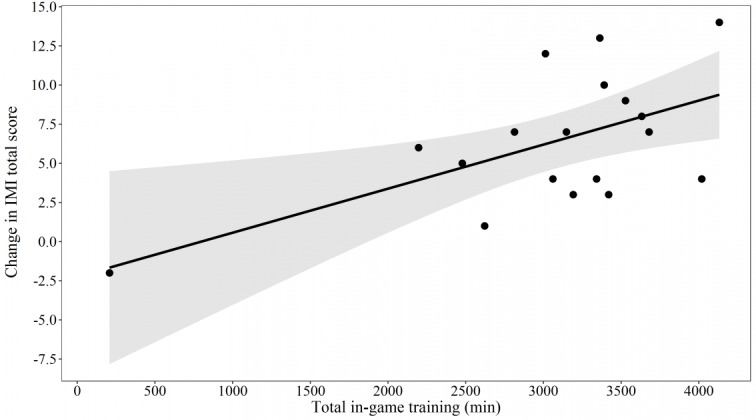
Linear regression model illustrating the relationship between total in-game training (minutes) and change in the Intrinsic Motivation Inventory total score. IMI: Intrinsic Motivation Inventory.

The linear regression model (Figure 4) showed a significant positive association between total in-game training (minutes) and change in IMI total score (beta=0.0028; 95% CI 0.0007-0.0049; *P*=.01; R^2^=0.34). This indicates that for every additional 30 minutes of in-game training per week during the 24-week intervention period, the total IMI score increased by 2.03 points (95% CI 0.54-3.51).

## Discussion

### Principal Findings

The results of this randomized controlled trial show that a novel smartphone game, whose storyline is based on established motivational elements and behavior change techniques, can significantly improve intrinsic PA motivation and lead to stable increases in PA in inactive patients with type 2 diabetes over a 24-week period. Participants in the intervention group showed significant increases in the IMI total score and the subscales “interest/enjoyment,”“perceived competence,” and “perceived choice,” indicating improvements in true PA-related intrinsic motivation and factors that predict intrinsic motivation. There was no significant change in the “value/usefulness” subscale in the intervention group; however, it is noteworthy that the baseline value of this intrinsic motivation-predicting subscale was relatively high (18.7 points) and thus did not leave a lot of room for improvement in a scale with a maximum score of 21 points. The average recorded time of 143 minutes of total in-game training per week during the intervention phase underlines the game’s strong potential in motivating formerly inactive patients with type 2 diabetes (39 minutes of moderate-to-vigorous PA per week at baseline) to meet and sustainably adhere to established PA recommendations [4]; this level also confirmed our hypothesis that the game-induced increases in intrinsic PA motivation would lead to an improved PA adherence.

### Interpretation of Results

Intrinsic PA motivation increased significantly in the intervention group during the 24-week intervention period. A comparison of the magnitude of these improvements with other studies is difficult, since no studies used IMI to assess changes in intrinsic motivation in game-based interventions that promote PA. However, a cross-sectional study [36] showed that enjoyment and perceived competence of performing a television exercise program (ie, higher intrinsic motivation) are the most predictive factors of more frequent participation in the program. The recorded in-game training data in our study support this finding. By illustrating the direct impact of the game-induced increase in intrinsic PA motivation on the weekly PA behavior, the usage data provide a more tangible meaning to the shown increase in the relatively abstract IMI scores, extending beyond the primarily predictive value of the IMI.

Analysis of the recorded usage data further shows that participants walked an average of 1893 steps per day during in-game training. This is distinctly higher than the amount reported for other PA-promoting smartphone games such as Pokémon GO, even when assuming that the reported maximal increase of 955 steps per day was entirely attributable to Pokémon GO-related walking [13]. Further, in contrast to Pokémon GO, which showed a gradual decline in daily steps back to baseline values after only 6 weeks of the abovementioned initial increase in daily PA, the amount of both in-game steps and overall recorded daily steps in our study was considerably more consistent. Average daily in-game steps between 1619 and 2206 throughout the intervention period indicate a substantially better PA adherence that is especially meaningful when considering the distinctly longer timeframe of 24 weeks. The stable, objectively measured increases in weekly PA during the intervention period, along with the positive association between total in-game training (minutes) and change in IMI score, further confirm the previously indicated [25,36] importance of pursuing improvements in intrinsic motivation through increased PA enjoyment especially in behavioral interventions targeting inactive and unmotivated individuals. As this smartphone game is a motivating and enjoyable experience, the subjectively perceived cost of PA is reduced and people are encouraged to engage in regular PA based on their personal intrinsic motivation [14,43]. Thus, our game’s narrative and gameplay may be more elaborate and the motivational elements incorporated into our storyline may be more successful in motivating players for long-term use as compared to Pokémon GO. It is further possible that our approach of tailoring the in-game PA recommendations and exercises to the fitness level of each player prevented feelings of incapability and failure that result from overwhelming PA volumes and intensities and instead enabled players to experience PA-related competence by allowing them to complete suitable workouts and meet appropriate and realistic PA goals. The effectiveness of our game is highlighted by the fact that 83.7% of all in-game walking (110 minutes/week) was of moderate-to-vigorous intensity (≥100 steps/minute) and therefore most likely suitable to improve aerobic fitness [44] and prevent morbidity and premature mortality [45]. This weekly amount of moderate-to-vigorous-intensity walking is equal to an average duration of 15.6 minutes per day, which has been shown to be associated with a 14% reduction in all-cause mortality and has therefore been proposed as the minimum amount of PA required to extend life expectancy [46].

The average amount of time spent in in-game strength exercises was distinctly lower than that spent in in-game walking throughout the intervention period. Although the completion rate of strength workouts was considerably lower than that of walking workouts (70.4% vs 96.5%), the shorter average engagement in strength workouts was a factor of how these workouts were designed. In contrast to walking workouts, which were designed to be of sufficient intensity and duration to improve aerobic fitness [4,44] while avoiding a demotivating physical overload, strength workouts were designed as brief bouts of activity to interrupt prolonged times of sedentary behavior that could be easily integrated into the daily routine and performed anywhere without extensive equipment. This design choice is supported by recent findings, showing that interrupting prolonged sitting by brief bouts (2-5 minutes) of PA every 20-30 minutes can yield improvements in glycemic control in inactive individuals with an impaired glucose regulation for up to 22 hours [47,48]. On an average, participants made use of this design feature approximately seven times per week, with an average duration of approximately 2.5 minutes per workout, and thereby made clinically relevant changes to their sedentary daily routine.

Overall, the game encouraged an average of 143 minutes of in-game activity per week and supported patients with type 2 diabetes who were physically inactive for many years, in adopting and adhering to a physically active lifestyle that corresponds to established PA guidelines [4].

### Limitations and Implications for Future Research

A limitation of this study is that we have no objectively measured record of any additional PA beyond the phone-recorded PA. Although participants used the game extensively as a training tool and accrued close to the guideline-recommended amount of 150 minutes of moderate-intensity PA per week during in-game training alone, it would have been interesting to see if participants engaged in any additional structured, moderate-intensity PA outside of the game. Further, although the phones recorded daily steps with a likely high accuracy when they were placed on the body [18], we have no record of the number of steps that were taken when the phones were not placed on the body. Although participants were encouraged to carry their phones with them as much as possible (ie, reminder function of the game), and based on the average daily step counts, it is likely that they did most of the time, it is quite conceivable that especially when participants were at home or work, they did not always carry their phones on them. These periods without phone wear likely led to an underestimation of unknown magnitude of the true number of daily steps. Therefore, future smartphone-based studies should consider measuring daily steps additionally with an accurate accelerometer that has the high potential for good wear-time compliance such as an activity wristband [18]. A further limitation is that we are not certain of which conceptual ideas and motivational elements incorporated into the game have indeed caused the increased intrinsic PA motivation and led to the improved PA behavior. Although it is justifiable to argue that the ensemble of all behavior change mechanics was crucial for the success of the game, a more detailed analysis would have provided important knowledge for future game designs. Finally, a follow-up assessment after an appropriate interim period should be considered to evaluate the effectiveness of the game regarding the sustainability of the improvements in PA adherence beyond the 24-week intervention period.

### Conclusions

In summary, our randomized controlled trial shows that a novel smartphone exergame that incorporates established motivational elements and personalized PA recommendations in the storyline can generate significant increases in intrinsic PA motivation in inactive individuals with type 2 diabetes. The clinical relevance of the game-induced increases in intrinsic PA motivation is highlighted by the associated *de facto* and stable improvements in PA adherence during the 24-week intervention period that demonstrate the game’s suitability for successfully encouraging persistently sedentary individuals to meet and adhere to established PA guidelines. The combination of playful elements and an individualized PA promotion has high potential for success in other inactive, unmotivated target groups with and without chronic diseases.

## Appendix

Multimedia Appendix 1 CONSORT‐EHEALTH checklist (V 1.6.1).

## Abbreviations

IMI: Intrinsic Motivation Inventory
IQR: interquartile range
PA: physical activity
